# Behavioral Economics and Tobacco Control: Current Practices and Future Opportunities

**DOI:** 10.3390/ijerph19138174

**Published:** 2022-07-04

**Authors:** Dalia Littman, Scott E. Sherman, Andrea B. Troxel, Elizabeth R. Stevens

**Affiliations:** 1 Department of Population Health, NYU Langone Health, New York, NY 10016, USA; dalia.littman@nyulangone.org (D.L.); scott.sherman@nyulangone.org (S.E.S.); abdrea.troxel@nyulangone.org (A.B.T.); 2 Department of Medicine, VA New York Harbor Healthcare System, New York, NY 10010, USA

**Keywords:** behavioral economics, tobacco, smoking, psychology, economics

## Abstract

Despite considerable progress, smoking remains the leading preventable cause of death in the United States. To address the considerable health and economic burden of tobacco use, the development of improved tobacco control and treatment interventions is critical. By combining elements of economics and psychology, behavioral economics provides a framework for novel solutions to treat smokers who have failed to quit with traditional smoking cessation interventions. The full range of behavioral economic principles, however, have not been widely utilized in the realm of tobacco control and treatment. Given the need for improved tobacco control and treatment, the limited use of other behavioral economic principles represents a substantial missed opportunity. For this reason, we sought to describe the principles of behavioral economics as they relate to tobacco control, highlight potential gaps in the behavioral economics tobacco research literature, and provide examples of potential interventions that use each principle.

## 1. Introduction

Improving tobacco control and treatment remain top healthcare priorities [1,2]. Despite considerable progress, smoking remains the leading preventable cause of death in the United States causing 480,000 deaths and USD 300 billion in health-related economic losses each year [3]. Currently available behavioral and medical treatments for smoking cessation have been used with only moderate success [4]. To address the considerable health and economic burden of tobacco use, there is a continuing need to develop improved tobacco control and treatment interventions.

By combining elements of economics and psychology, behavioral economics provides a framework for designing novel solutions to treat smokers who have failed to quit smoking with traditional smoking cessation interventions. Behavioral economics uses variants of traditional economic assumptions to understand how and why people behave the way they do in the real world. Principles from behavioral economics research have been successfully used to develop policy frameworks and as the foundation for health interventions to encourage people to make healthful choices in other fields of medicine [5,6]. In one framework of behavioral economics, Dawnay and Shah identified seven principles including (1) other people’s behavior matters; (2) habits are important; (3) people are motivated to ‘do the right thing’; (4) self-expectations influence behavior; (5) people are loss-averse; (6) people are bad at computation; (7) people want to feel involved and efficacious [7]. The full range of these principles, however, has not been widely utilized in the realm of tobacco control and treatment.

The vast majority of tobacco-related studies using behavioral economics have focused on financial incentives and/or competition [5,8,9,10,11], and few studies have explored other behavioral economic principles. Insufficient evidence severely limits the ability of decision makers (i.e., policy makers and program directors) to determine the appropriateness of behavioral economic approaches to tobacco control and treatment. Therefore, we propose expanding the potential application of behavioral economics principles for tobacco control and treatment. Given the need for improved tobacco control and treatment, the limited use of other behavioral economic principles represents a considerable missed opportunity. For this reason, we sought to describe the principles of behavioral economics as they relate to tobacco control, highlight potential gaps in the behavioral economics tobacco research literature, and provide hypothetical examples of potential interventions that use each principle.

### Gaps in the Behaviroal Economics Tobacco Literature

To highlight specific gaps in the behavioral economics tobacco control literature and augment the anecdotal perception that these gaps exist, we performed a rapid scoping survey of the primary research. Our survey of the literature was not an exhaustive review, but we sought to answer the question: What tobacco control and treatment research has been performed with a basis in behavioral economic principles? Our search strategy sought to capture research within the broad scope of behavioral economics and account for the limited labeling of this research as ‘behavioral economics’. In addition to terms associated with tobacco use, search terms used included a robust, but not exhaustive, list of terms associated with behavioral economic principles (e.g., contingency management, nudging, contract, incentive etc.). Searches were conducted in March 2021 using MEDLINE (PubMed), and PsychINFO (OVID), with Google Scholar used as a supplement to identify potential research from other disciplines not indexed on the other platforms. Title and abstract review were performed in duplicate by two authors (ERS and DL), with discrepancies resolved via discussion. Article summarization was not performed in duplicate, but the identified behavioral economic principle was confirmed by the second author.

A total of 230 primary research articles were identified, and data were collected for behavioral economic focus, study population, sample size, and primary outcome. Of the identified studies, 198 pertained to the use of financial incentives and/or contingency management or financial demand; the other behavioral economic topics examined included: principle 1 in the form of competition (8) [12,13,14,15,16,17,18,19]; principle 2 as reinforcement/nudging (7) [20,21,22,23,24,25,26] and replacement (5) [27,28,29,30,31,32]; principle 4 with behavioral contracts (2) [33,34]; principle 5 by means of deposit contracts (5) [35,36,37,38,39]; principle 6 via exploration of discounting (4) [40,41,42,43] and prospect theory (1) [44]. The seven principles of behavioral economics were used to synthesize the literature to identify gaps in the research (presented in the Table 1, excluding financial demand and financial incentives/contingency management).

## 2. Seven Principles of Behavioral Economics

Neoclassical economics assumes that humans are rational and behave in a way to maximize their individual self-interest. This “rational economic person” assumption, however, has had limited success in predicting human behavior. Rooted in psychology, behavioral economic theory is used to explore views of human nature and decision making that exceed the simple axioms of the neoclassical model. The seven previously discussed principles of behavior economic theory [7] identify patterns of human behavior, and can be leveraged to develop improved tobacco control and treatment interventions and policy.

With the aim of further exploring the role of behavioral economics in tobacco control and treatment, we used these seven principles of behavioral economic theory as an analytical framework to highlight areas that have not yet received meaningful attention in the tobacco control and treatment literature. We then outlined how each principle may apply to tobacco control and treatment, and described the currently available studies as well as hypothetical interventions based upon each principle (presented in Table 1).

### 2.1. Principle 1: Other People’s Behavior Matters

When making choices, people tend to model their own behavior on that of those around them. Due to the impossibility of rationally obtaining and assessing all behavioral options for a given choice, people implicitly use observations from their immediate context to determine prevalent social norms. The extent of the influence of others’ behavior is partially driven by who the “influencer” is. In-group bias suggests a predilection for modeling the behavior of those with a shared social identity. In trials of competitions as a mechanism for smoking cessation, those that were based in workplaces were consistently effective, highlighting the importance of normalizing a behavior within an in-group in order to drive behavioral change [45]. The influence of celebrities on health behaviors has been demonstrated in the past, most notably the encouragement of colonoscopies by Katie Couric and BRCA genetic testing by Angelina Jolie [46,47]. In tobacco control, this principle has been explored in the form of peer-focused interventions such as the European program “Be smart, don’t start” [15,16,17,18,19] and workplace competitions [12,13,14].

The impact of others’ behavior on one’s own choices presents numerous opportunities to intervene and promote smoking cessation. Celebrities and online influencers have demonstrated the ability to drive the actions of followers. Therefore, future research can assess interventions such as the following: (1) campaigns from influencers who themselves have quit smoking about the process and its outcomes, which may encourage viewers to follow a similar path; (2) given the impact of in-groups on behavior, quit interventions run through groups of colleagues, religious community members, or designated “quit buddies” may drive higher rates of cessation.

### 2.2. Principle 2: Habits Are Important

Behavior is habitual, with the same routines performed daily. Relying on habits reduces the mental energy required to complete tasks, even if habits are not efficient or healthy. Changing habits requires both stopping current actions and completing new actions, which, though difficult to accomplish, has the potential for ensuring long-lasting change. Bans on smoking in bars and offices put in place by the Clean Indoor Air Act in New York City in 2003 forced smokers out of their habits of smoking in such locations, resulting in a decline in smoking rates and lower exposure to secondhand smoke within 3 years of passage [48]. A study of people who successfully quit smoking found that those who changed their routines, e.g., by including more exercise, were more likely to maintain abstinence [32].

Habits provide opportunities for behavioral change through two primary methods: taking advantage of current habits and creating new habits. Future research may assess whether replacing physical cues for cigarettes leads to a decline in cigarette use. Example interventions include providing alternatives to cigarettes that can be used in place of smoking (e.g., harm reduction with e-cigarettes) to decrease the likelihood of relapse.

### 2.3. Principle 3: People Are Motivated to ‘Do the Right Thing’

The perception that an action is completed for the public good or for the good of others influences behavior. The inverse of this principle was also demonstrated: paying people to donate blood results in a reduced number of blood donors in long term [49]. Appealing to people’s sense of altruism can be an effective and efficient approach to behavior change. Smoking cessation interventions leveraging an individual’s motivation to ‘do the right thing’ have mostly focused on public messaging campaigns and interventions designed to emphasize the importance of quitting to protect children and others from secondhand smoke [50]. Tobacco companies fought the evidence on secondhand smoke for many years because of clear evidence that it was an effective motivator for smokers to quit [51].

Intrinsic motivation can be derived from activities such as cooperative teamwork, as responsibility toward the well-being of others can also outweigh a sense of responsibility toward oneself [52]. Cooperating to achieve a goal, rather than achieving a goal by oneself, can increase intrinsic motivation. Therefore, future research may develop a program that creates teams to collectively achieve a smoking cessation goal, potentially improving motivation to quit. An example intervention may include teams of two or more smokers who receive an incentive if all team members achieve defined goals such as starting medication, attending counseling, or maintaining abstinence.

### 2.4. Principle 4: Self-Expectations Influence Behavior

Individuals want their actions to be in line with their values and commitments. Discrepancy between actions and beliefs may lead to individuals changing their beliefs to fit their behaviors. When beliefs are expressed openly, however, people are more likely to change their behavior to remain consistent with those expressed beliefs. Openly committing to friends, family, and even strangers can increase the extent to which behavior change occurs. Eliciting promises and signed pledges, even when there is no direct consequence of breaching the contract, has been shown to affect behavior in a variety of settings, from cheating to seatbelt wearing [53,54,55,56]. In one small trial (*n* = 28), behavioral contracting was used with some success to enhance smoking cessation maintenance [34].

To leverage this principle, in future research, a scenario can be created in which quitting smoking is an expectation, using a pledge that participants must sign and announce to others, perhaps also reading it aloud each day. Based upon the behavioral economics literature, this seemingly minor intervention with minimal associated risk may have an unexpectedly large effect as individuals attempt to match their actions to their words.

### 2.5. Principle 5: People Are Loss-Averse

Behavioral economics research suggests that losses produce more emotional reactions than comparably-sized gains. Smoking cessation research featuring financial consequences, however, primarily focused on the potential for monetary gain rather than the potential for loss [9]. Such financial incentives were shown to increase the likelihood of smoking cessation [9]. Thus, developing and testing interventions that change the framing of incentives to exploit the principle of loss aversion, as opposed to gain alone, may further enhance the effectiveness of financial consequence-based interventions on smoking cessation. In tobacco control and treatment, however, the potential use of loss aversion has only been tested to a small extent in the form of deposit contracts [35,36,37,38,39].

One potential intervention that employs the principle of loss aversion is to offer a set amount of money to all who complete a smoking cessation program, and to deduct money for each check-in after a predetermined cessation date at which participants are not abstinent. This inverts the framing of incentives from potential gain to potential loss [57]. Although not deeply explored, the role of deposit contracts, where money is perceived as lost rather than gained, has produced higher sustained smoking abstinence than usual care [58] and may be cost-effective [38]. Similar interventions have shown promise in the areas of medication adherence [59,60] and physician quality and safety behaviors [61,62].

### 2.6. Principle 6: People Are Bad at Computation

Many studies suggest that humans poorly understand probability, overweighing small probabilities and putting more weight on the current state while discounting future outcomes [63,64,65,66]. These findings help explain the continued popularity of lotteries [28,29] and the difficulties dieters face in abstaining from readily available desserts. Quitting smoking involves several issues related to computation, including the difficulty in comprehending the associated health risks and balancing the near-term rewards of using nicotine against the long-term health rewards of quitting.

To take advantage of the appeal of lotteries, a “regret” lottery [67,68] can be run in which smokers are told what they *would have won* had they completed the required action (i.e., abstaining or using medications for cessation) if they fail to complete an assigned task. In addition, varying the size of both the chance of winning and the award can maintain interest, reduce predictability, and capitalize on gamification [69,70,71].

### 2.7. Principle 7: People Want to Feel Involved and Efficacious

Classic economic theory posits that giving people more choices results in better decisions. In reality, more choices are beneficial only up to a point after which expanding the realm of possibilities becomes overwhelming. Balancing these concepts, increasing self-efficacy by helping smokers to understand their options for quitting smoking, and emphasizing their capability to do so, makes people more likely to quit smoking [72].

Therefore, future research may assess the impact of providing choice in smoking cessation. One approach that merits testing is whether allowing people to choose a smoking cessation intervention from a selection of evidence-based approaches makes them more likely to quit. The ongoing FIESTA II study is using a similar strategy, comparing goal-directed financial incentives (paying people to use evidence-based smoking cessation treatment) with outcome-based incentives (paying people to be abstinent at different time points) [73]. Prior to randomization, participants were asked which approach they prefer. The data from this study will provide insight into whether people are more likely to quit if they receive their preferred intervention approach.

## 3. Discussion

Despite significant progress in tobacco control and treatment, current standard-of-care treatments, including behavioral counseling and nicotine replacement therapy (NRT), show only a modest, albeit significant, impact on the success of long-term smoking cessation [4]. Even with standard cessation treatment, engagement in cessation interventions is often low. There is a continuing need to develop improved interventions targeting tobacco use. Applying behavioral economics principles to current evidence-based practices holds considerable potential for informing innovative solutions to address the persistent use of tobacco products. In this work, we outlined the how seven principles of behavioral economics relate to tobacco use and presented examples of interventions that can be the target of future research within each principle.

The primary focus of most studies examining the role of economics in tobacco use has involved leveraging concepts of financial incentives and/or competition [5,8,9,10,11]. Although financial incentives have some impact on smoking cessation, they tend to appeal more to the tenets of neoclassical economics and do not consider the often seemingly irrational aspects of human behavior. The use of behavioral economics has the potential to further increase the impact of economic approaches to tobacco control and treatment by taking advantage of predictable human idiosyncrasies.

Despite its potential, however, behavioral economics and its various principles have not been well-explored in the field of tobacco control and treatment. Although objectively limited by not being systematic or entirely exhaustive, meaning we may have missed some articles, in our survey of the literature, we identified only four randomized controlled trials that examined behavioral economic principles outside of financial incentives and competition. Two studies used the loss aversion principle [38,39], one used social influence [16], and one used the self-expectations principle [34]. Of the few studies that have been performed, most had exceedingly small sample sizes, limiting their power and generalizability.

All seven principles have large gaps in the literature, with only principles 1 (other people’s behavior), 2 (habits), and 5 (people are loss adverse) having any randomized controlled study. However, even these principles do not contain sufficient available literature in tobacco control. The limited quantity and quality of behavioral economics research in these areas considerably restricts the ability to make informed decisions regarding its use in tobacco policy and program development. Given the hundreds of economics-focused tobacco studies and the thousands more of otherwise-focused tobacco control and treatment studies, the lack of behavioral economic research is disconcerting and represents a potentially serious missed opportunity.

This is particularly striking because use of these behavioral economic principles has been shown to significantly impact health behaviors in other fields. Previous researchers have used tactics such as gamification to overcome computational errors in medication adherence and weight loss [69,70,71]; deposit contracts to take advantage of loss aversion in medication adherence [59,60], and physician quality and safety behaviors [61,62]; and behavioral contracts to set self-expectations for seatbelt wearing [53]. These same principles, among others, are translatable to smoking cessation interventions, and can be incorporated into evidence-based practices. There are numerous behavioral economics-principle-based interventions that may be effective to supplement currently available tobacco treatments. The example lines of inquiry presented here are a small fraction of the possible behavioral economic research topics that can be pursued.

## 4. Conclusions

Behavioral economics principles offer potential strategies to enhance the current standard of care for tobacco treatment. Research leveraging these principles for tobacco treatment, however, has been limited. Given the trajectory of the success of currently available tobacco treatments, not exploring this field represents a significant missed opportunity. Researchers and funders alike should consider focusing on this area.

## Figures and Tables

**Table 1:** Available studies and hypothetical examples of tobacco interventions by behavioral economic principle.

*Hypothetical Example*		*Available Studies and Sample Size*
***1. Other people’s behavior matters***		
*Influencer campaigns*	Produce campaigns from influencers who themselves have quit smoking about the process and its outcomes	Klesges (1987) *n* = 136 [12]; Cummings (1988) *n* = 354 [13]; Sloan (1990) *n* = 73 [14]; Wiborg (2002) *n* = 2142 [15]; Crone (2003) *n* = 2562 [16]; Schneider (2006) *n*= 4358 [17]; Hanewinkel (2007) *n* = 12,812 [18]; Schulze (2006) *n* = 1704 [19].
*Social group quitting*	Run quit interventions through groups of colleagues, religious community members, or designated “quit buddies”	
*Learning from others*	Counsel patients on how many others use smoking cessation medications to increase acceptance	
***2. Habits are important***		
*Cue replacement*	Package nicotine replacement therapy in cigarette boxes and place those boxes where someone normally keeps cigarettes	Epstein, (1991) *n* = 8 [20]; DeGrandpre (1994) *n* = 6 [27]; Bickle (1997) *n* = 4 [30]; Tidey (1999) *n* = 6 [21]; Shahan (2000) *n* = 8 [28]; Johnson (2003) *n* = 11 [22]; Audrain-McGovern (2004) *n* = 983 [23]; Leventhal (2014) *n* = 275 [24]; Goelz (2014) *n* = 469 [25]; Murphy (2016) *n* = 86 [26]; Snider (2017) *n* = 385 [29]; Peters (2017) *n* = 82 [31]; Sohlberg (2020) *n* = 705 [32].
*Behavior replacement*	Provide alternatives to cigarettes that mimic the smoking experience people can use at the time they normally smoke, e.g., e-cigarettes	
*Default Bias*	Provide smoking counseling to all patients unless they opt out	
***3. People are motivated to ‘do the right thing’***		
*Collective goals*	Create teams to collectively achieve a smoking cessation goal, e.g., teams of two or more smokers who receive an incentive if all team members achieve defined goals such as starting medication, attending counseling, or maintaining abstinence	No studies identified
*Promises to others*	Have the participant make a pledge directly to a family member/close friend who will then participate in formal check-ins	
***4. People’s self-expectations influence how they behave***		
*Quitting pledge*	Create a scenario in which quitting smoking is an expectation using a pledge that participants must sign and announce to others	Singh (1988) *n* = 7 [33]; Bowers (1987) *n* = 28 [34].
*Setting expectations*	Have smokers set an expected smoking target, such as a number reduced of cigarettes per day, and have participants keep a log of their smoking to compare	
***5. People are loss-averse and hang on to what they consider ‘theirs’***		
*Incentive deduction*	Offer a set amount of money to all who complete a smoking cessation program and deduct money for each check-in after a predetermined cessation date at which participants are not abstinent	Winett (1973) *n* = 45 [35]; Paxton (1980) *n* = 60 [36]; Paxton (1983) *n* = 60 [37]; Toll (2007) *n* = 258 [39]; Dallery (2008) *n* = 8 [38].
*Deposit loss*	Have a patient have a buy-in that is returned only if goals are met	
***6. People are bad at computation***		
*Regret lottery*	Run a “regret” lottery in which smokers are told what they would have won had they completed the required action (e.g., abstaining or using medications for cessation) if they fail to complete an assigned task	Ohmura (2005) *n* = 50 [40]; Field (2006) *n* = 30 [41]; Sheffer (2012) *n* = 97 [42]; MacKillop (2012) *n* = 13 [43]; Leone (2015) *n* = 42 [44].
*Immediate reward*	Give rewards at the time of completing a task, rather than at the end of an intervention	
***7. People need to feel involved and effective to make a change***		
*Intervention choice*	Allow smokers to choose a smoking cessation intervention from a selection of evidence-based approaches	No studies identified
*Quit date choice*	Allow smokers to choose and set their own smoking quit date	

## Data Availability

Not applicable.
